# Prediction of labour onset in women who present with symptoms of preterm labour using cervical length

**DOI:** 10.1186/s12884-021-03828-z

**Published:** 2021-05-05

**Authors:** Tiffany Tuck Chin Wong, Xiaoqi Yong, Janice Su Zhen Tung, Beatrice Jia Ying Lee, Joanne Mei Xin Chan, Ruochen Du, Tai Wai Yeo, George Seow Heong Yeo

**Affiliations:** 1 Department of Obstetrics and Gynaecology, KK Women’s and Children’s Hospital, 100 Bukit Timah Road, 229899 Singapore, Singapore; 2 Department of Maternal Fetal Medicine, KK Women’s and Children’s Hospital, Singapore, Singapore

**Keywords:** Preterm labour, Transvaginal ultrasound, Cervical length, Prediction of preterm labour, Threatened preterm labour

## Abstract

**Background:**

Diagnosis of preterm labour is difficult because initial symptoms and signs are often mild and may occur in continuing pregnancies. This study aims to investigate the utility of measuring cervical length, using transvaginal ultrasound, in women presenting to the delivery suite with symptoms of preterm labour.

**Methods:**

This was a prospective cohort study performed in KK Women’s and Children’s Hospital, Singapore from September 2017 to July 2018. Women with singleton pregnancies, presenting with symptoms of contraction pain, between 24^+ 0^ to 36^+ 6^ weeks gestation, were included. Transvaginal ultrasound cervical length measurements were done at presentation to the labour ward, after four hours and in the following morning. The primary outcome of the study was delivery within 1 week. All statistical analyses were conducted with Microsoft Excel and Statistical Package for the Social Sciences.

**Results:**

A total of 95 subjects were included. A one-millimeter increase in the 1st cervical length increases scan-to-delivery time by 0.802 days (*p*-value 0.003, CI 0.280–1.323). Receiver Operator Characteristic (ROC) curve analysis for prediction of delivery within 1 week showed an Area Under Curve (AUC) of 0.667, optimal cut-off value of 27.5mm (sensitivity 77.8 %, specificity 61.6 %). A one-millimetre increase in the 3rd cervical length increases scan-to-delivery time by 0.770 days (*p*-value 0.023, CI 0.108–1.432). ROC curve analysis for prediction of delivery within 1 week showed an AUC of 0.915, optimal cut-off value of 25.5mm (sensitivity 100 %, specificity 73.6 %). However, the change in cervical length over a period of 1 day was not significant in predicting delivery within 1 week.

**Conclusions:**

Our results indicate that by using a cervical length cut off of 27.5mm at presentation, we would have predicted 77.8 % of deliveries within 1 week. If we were to repeat the cervical length scan the next day, with the same cut-off of 27.5mm, we would have predicted 100 % of deliveries within 1 week. In our study, measuring the transvaginal ultrasound cervical length is a reliable diagnostic test for delivery within 1 week. However, the results are limited by the small sample size. Further studies should be conducted with a larger sample size.

## Background

Preterm birth is defined as birth before 37 weeks’ gestation. The estimated global preterm birth rate for 2014 was 10.6 %, equating to an estimated 14.84 million live preterm births [1]. It is the leading cause of neonatal morbidity and mortality in most countries. Initiation of interventions to improve neonatal outcomes such as antenatal corticosteroids, group B streptococcal infection prophylaxis, and magnesium sulphate for neuroprotection are often dependent on the precise time of delivery, making prediction of labour onset important.

Diagnosis of labour onset is difficult because initial symptoms and signs are often mild and may occur in continuing pregnancies [2]. Less than 10 % of women with a clinical diagnosis of preterm labour (PTL) will deliver within seven days of initial presentation [3]. Various biochemical tests have been studied for the early detection of labour. However, literature describing the use of ultrasound in the early detection of labour is scarce. Currently, there is not enough high-quality research to show if knowledge of cervical length in women has any effect [4].

Ultrasound cervical shortening can be detected weeks before delivery, at about 32 weeks for term births and as early as 16–24 weeks for preterm births [2]. A study conducted on 934 asymptomatic patients who had serial cervical length measurements performed at 4 antenatal visits showed that there is a significantly shorter cervical length in the 2nd and 3rd trimester in patients who eventually had a preterm birth [5]. However, the role for cervical length measurements in women with symptoms of PTL remains unclear. A Cochrane review (2019) conducted on 4 randomised controlled trials, which examined knowledge of transvaginal ultrasound-measured cervical length of singletons with symptoms of PTL versus no knowledge, concluded that they are uncertain of the effects because of inconclusive results and very low-quality evidence for preterm births at less than 37 weeks [4].

This study aims to investigate the utility of measuring cervical length, using transvaginal ultrasound (TVUS), in women presenting with symptoms of PTL. We are studying if the (i) cervical length at presentation and (ii) the change in the cervical length over a period of one day, can predict delivery within 1 week.

## Methods

This is a prospective cohort study performed in KK Women’s and Children’s Hospital, Singapore from September 2017 to July 2018. Women with singleton pregnancies, presenting with symptoms of contraction pain, between 24^+ 0^ to 36^+ 6^ weeks gestation, were recruited into the study. Contraction pain was defined as painful tightening or hardening of the uterus that comes in waves, and these contractions were recordable on cardiotocography. Subjects who had dropped out of the study before commencing on any cervical length measurements, lost to follow-up, or had iatrogenic preterm births, were excluded from the study. Informed consent was taken from the participants before the commencement of the study.

Cervical length measurements were done at presentation to the labour ward, after four hours and in the following morning. These measurements were done via the TVUS method and were performed by 3 designated sonographers who had extensive experience and accreditation by the Fetal Medicine Foundation for competency in performing this scan. The scans were done in line with the standards set by the Fetal Medicine Foundation [6]. Each examination was performed over a period of 3 min and the best shortest measurement of cervical length was recorded.

We followed-up these subjects to delivery with the primary outcome of the study being delivery within 1 week. The secondary outcomes were scan-to-delivery time and delivery within 2 weeks. Other data collected included demographics, presenting symptoms and signs, and risk factors for PTL (previous mid-trimester miscarriage, previous preterm birth, previous uterine evacuations, previous cervical surgery, genitourinary infections, periodontal disease, short inter-pregnancy interval, smoking, alcohol consumption, substance abuse).

All statistical analyses were conducted with Microsoft Excel and Statistical Package for the Social Sciences. Fisher’s exact test was used for categorical variables, Student’s t-test and Mann-Whitney U test for continuous variables. Univariate and multivariate linear regressions were performed to assess the correlation between cervical length and scan-to-delivery time. Receiver Operator Characteristic (ROC) curves were also performed to determine the optimal cut-offs of cervical length that can predict delivery within 1 week. The Area Under Curve (AUC) were calculated to assess the performance of the ROC curves. Statistical significance was defined to be *p*-value of < 0.05.

 Ethics approval for this study was obtained from the Singhealth Centralised Institutional Review Board (reference number 2016/3060). This study was funded by the Singhealth Duke-NUS OBGYN Academic Clinical Program Research Grant 2017.

## Results

### Demographics

We recruited a total of 107 subjects who presented with symptoms of contraction pain. Four subjects were excluded as they had an iatrogenic preterm delivery – 2 for scar dehiscence, 1 for placental abruptio, and 1 for severe intrauterine growth restriction. Six subjects had dropped out of the study before commencing on any scans, and 2 subjects were lost to follow-up. Of the remaining 95 subjects included in the analysis, the mean body mass index was 25.4, the mean age was 29.3 years old and the mean gestational age at presentation was 33.5 weeks. Ethnically 28.4 % were Chinese, 56.8 % were Malay and 8.4 % were Indian. There were 30 nulliparous women and 65 multiparous women. Table 1 shows the risk factors for preterm birth of the study population, while Table 2 shows the clinical signs of the subjects on presentation to the labour ward.

**Table 1:** Risk factors for preterm birth of study population

Risk factor	*N* (%)
Previous mid-trimester miscarriage	2 (2.1)
Previous preterm delivery	24 (25.3)
Previous uterine evacuation	30 (31.6)
Previous cervical surgery	1 (1.1)
Genitourinary infections in current pregnancy	58 (61.1)
Periodontal disease	0 (0.0)
Inter-pregnancy interval < 6 months	2 (2.1)
Smoking	10 (10.5)
Alcohol consumption	4 (4.2)
Substance abuse	0 (0.0)

**Table 2:** Relationship between risk factors and presenting clinical signs with delivery within 1 week

	Delivered within 1 week		
	**Yes** *N*** = 9**	**No** *N*** = 86**	*p***value**
**Risk factors**			
** Previous mid-trimester miscarriage**			
Yes No	0 9	2 84	1.000
** Previous preterm birth**			
Yes No	4 5	20 66	0.224
** Previous uterine evacuations**			
Yes No	3 6	27 59	1.000
** Genitourinary infections**			
Yes No	6 3	52 34	1.000
** Smoker**			
Yes No	0 9	10 76	0.590
** Alcohol consumption**			
Yes No	0 9	4 82	1.000
** Clinical signs**			
** Uterine activity on cardiotocography**			
Regular contractions Irregular contractions	8 1	52 34	0.147
** Cervical dilatation**			
Cervix ≥ 3 cm Cervix < 3 cm	1 8	3 82	0.336
** Cervical effacement**			
Effaced Not effaced	1 8	7 78	0.568
** Actim partus test**			
Positive test Negative test	3 1	13 34	0.086

**Fig. 1:**
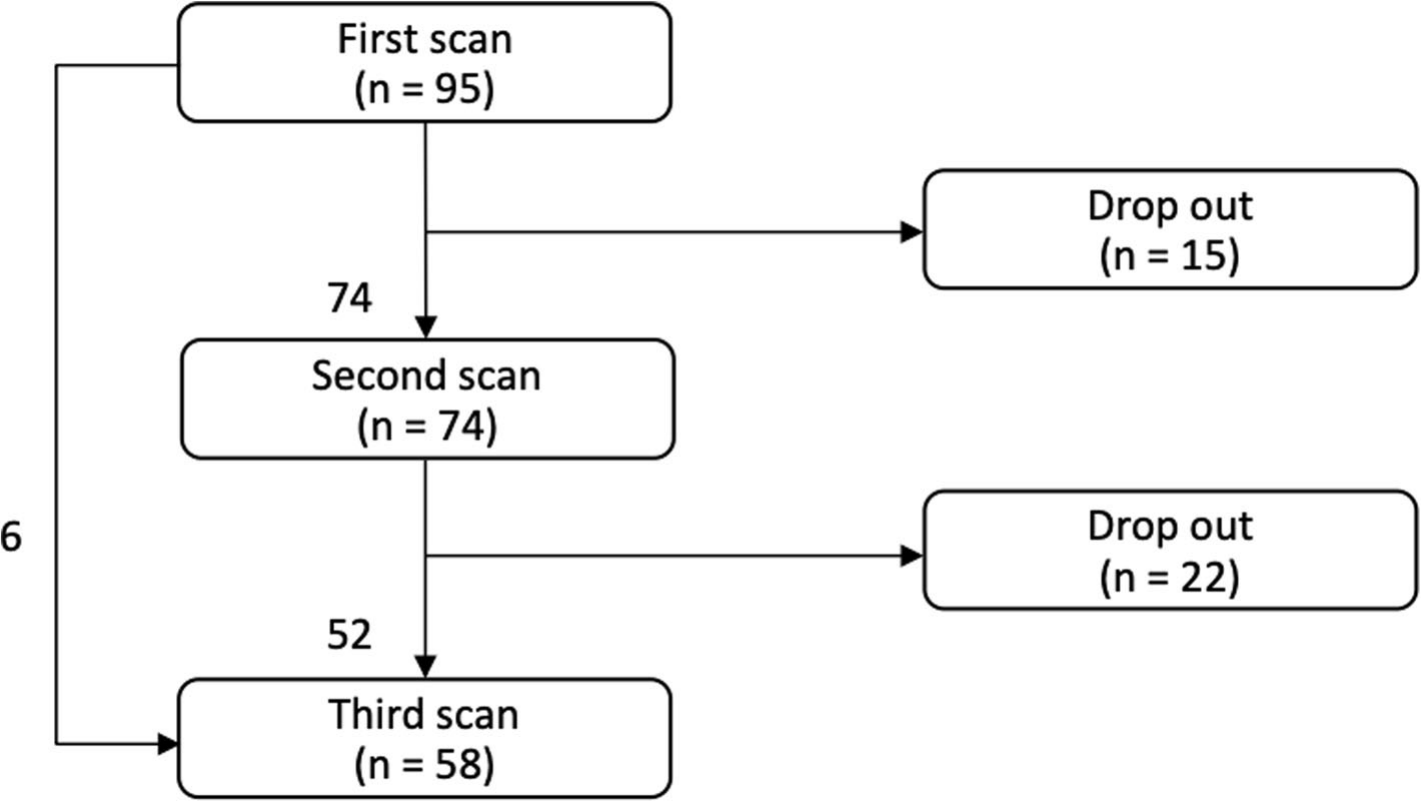
Overview of subjects flow

All 95 subjects had at least 1 cervical length scan done, 74 subjects had 2 cervical length scans done and 52 subjects had completed all 3 cervical length scans (Fig. 1). Reasons for missing scans include being discharged from the hospital, required pain relief in the labour ward, had per-vaginal bleeding or had declined further scans. None of the subjects had delivered before the completion of scans.

### Outcomes

There were 9 subjects (9.5 %) who delivered within 1 week of presentation, and 15 subjects (15.8 %) who delivered within 2 weeks of presentation (Table 3). The preterm birth rate of study population was 14.7 %. Using Fisher’s exact test, risk factors and clinical signs were not significantly different between the women who had delivered within 1 week and after 1 week (Table 2). Sixteen women had a positive Actim Partus test, of whom only 3 had delivered within 1 week.

**Table 3:** Number of subjects who delivered within 1 week or 2 weeks

	*N*	Delivery within 1 week	Delivery within 2 weeks
**Subjects who underwent 1st cervical length scan = total study population**	95	9	15
**Subjects who underwent 2nd cervical length scan**	74	7	12
**Subjects who underwent 3rd cervical length scan**	58	5	7

**Fig. 2:**
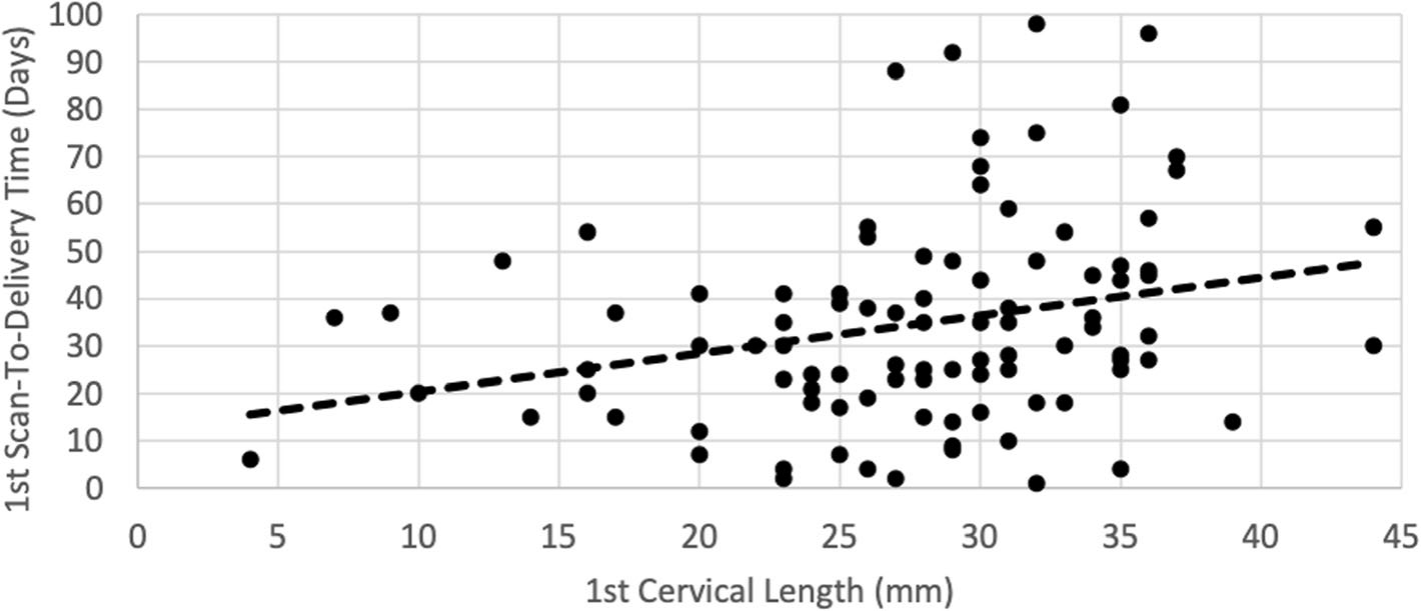
Correlation between 1^st^ cervical length and scan-to-delivery time (days)

**Fig. 3:**
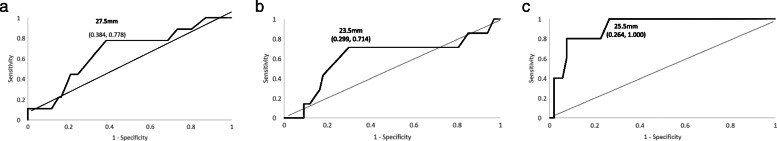
**a**, **b**, **c** (from left to right): ROC curve for the 1st, 2nd and 3rd cervical length respectively, as a predictor of delivery within 1 week

There was a positive correlation between the 1st cervical length with scan-to-delivery time (Fig. 2). By multiple linear regression, a one-millimeter increase in cervical length increases the scan-to-delivery time by 0.802 days (p-value 0.003, CI 0.280–1.323) (after adjusting for parity and gestational age). Out of the 95 subjects who had the 1st cervical length scan done, 9 subjects delivered within 1 week. On the ROC curve analysis, it showed an AUC of 0.667 for the prediction of delivery within 1 week, with the optimal cut-off value of 27.5mm (sensitivity 77.8 %, specificity 61.6 %) (Fig. 3a).

However, the positive correlation between the 2nd cervical length with scan-to-delivery time was not statistically significant. Out of the 74 subjects who had the 2nd cervical length scan done, 7 subjects delivered within 1 week. On the ROC curve analysis, it showed an AUC of 0.619 for the prediction of delivery within 1 week, with the optimal cut-off value of 23.5mm (sensitivity 71.4 %, specificity 70.1 %) (Fig. 3b).

There was also a significant positive correlation between the 3rd cervical length with scan-to-delivery time. By multiple linear regression, a one-millimeter increase in cervical length increases the scan-to-delivery time by 0.770 days (p-value 0.023, CI 0.108–1.432) (after adjusting for parity and gestational age). Out of the 58 subjects who had the 3rd cervical length scan done, 5 subjects delivered within 1 week. On the ROC curve analysis, it showed an AUC of 0.915 for the prediction of delivery within 1 week, with the optimal cut-off value of 25.5mm (sensitivity 100 %, specificity 73.6 %) (Fig. 3c).

ROC curve analyses were performed for each cervical length for the prediction of delivery within 2 weeks. However, the AUC of the ROC curves were close to 0.5.

A sub-group analysis of the nulliparous population (30 subjects) also showed similar results, with a positive correlation between each cervical length and the scan-to-delivery time. However, this was only statistically significant on the 1st cervical length. By simple linear regression, a one-millimetre increase in cervical length increases the scan-to-delivery time by 0.377 days (*p*-value 0.040, CI 0.084–3.403). On the ROC curve analysis, it showed an AUC of 0.813 for the prediction of delivery within 1 week, with the optimal cut-off value of 26mm (sensitivity 100 %, specificity 67.9 %). ROC curve analysis of the 2nd cervical length showed an AUC of 0.855 for the prediction of delivery within 1 week, with the optimal cut-off value of 24mm (sensitivity 100 %, specificity 73.7 %). ROC curve analysis of the 3rd cervical length showed an AUC of 1 for the prediction of delivery within 1 week, with the optimal cut-off value of 21mm (sensitivity 100 %, specificity 100 %).

We subsequently calculated the absolute and percentage change in cervical lengths for each subject, between the 1st and 2nd scan, and between the 1st and 3rd scan. The absolute change was calculated by subtraction of the 2nd cervical length from the 1st cervical length and the subtraction of the 3rd cervical length from the 1st cervical length. The percentage change was calculated by the subtraction of the 2nd cervical length from the 1st cervical length, divided by the 1st cervical length, and the subtraction of the 3rd cervical length from the 1st cervical length, divided by the 1st cervical length. With Mann Whitney U test, the absolute and percentage change were not significantly related to outcomes of delivery within 1 or 2 weeks.

## Discussion

The diagnosis of onset of labour is imprecise and it has particular importance in preterm pregnancies. The traditional presentation of preterm labour (regular uterine contractions accompanied by progressive cervical dilatation and effacement) are most diagnostic when contraction frequency is six or more per hour, cervical dilatation is 3 cm or more, effacement is 80 % or more, membranes rupture, or when bleeding occurs [2, 7, 8]. However, these signs often occur too late to allow effective intervention [9]. Most women with threatened PTL have minimal cervical dilatation (≤ 2 cm) on manual examination and about 75 % do not deliver preterm [10, 11]. Therefore, in these women, cervical length on TVUS had been suggested as a better screening tool to determine the need for intervention [11]. Ultrasound assessment of the cervical length are standardised, objective imaging often done by an independent body, with measurements documented and achieved for re-assessment. Digital assessment by a clinician suffers from subjectivity, lack of documentary proof and cannot be achieved for re-assessment.

In our study, we have found that cervical length is an accurate predictor of delivery within 1 week. In women who present with symptoms of PTL, by using a cervical length cut off of 27.5mm at presentation, we would have predicted 77.8 % of deliveries within 1 week, with a false positive rate of 38.4 %. If we were to repeat the cervical length scan the next day, with the same cut-off of 27.5mm, we would have predicted 100 % of deliveries within 1 week with a false positive rate of 34.0 %. When we further analysed the nulliparous population, the AUC of the ROC curves were even closer to 1, which substantiated our findings of using cervical length as an accurate predictor of delivery within 1 week. In this nulliparous population, using the same 27.5mm cut-off for either of the 3 cervical lengths, would identify all patients who delivered within 1 week.

Previous studies have also suggested similar cervical length cut-offs. Sotiriadis et al. performed a meta-analysis and found that the cervical length cut-off of < 20mm could predict 75.4 % of preterm births within one week, with a specificity of 79.6 %, while the cervical length cut-off of 25mm had a sensitivity of 78.3 % and a specificity of 70.8 % [12]. However, as the cervical length is known to change with gestation, Palacio et al. suggested that different cervical length cut-offs should be used at different gestational ages, with a cut-off of 25mm in women presenting with threatened PTL at < 32 weeks’ gestation but a cut-off of 15mm in women presenting at >/= 32 weeks’ gestation [13].

However, there have been conflicting evidence on the value of serial cervical length measurements in symptomatic women. A prospective study by Sotiriadis et al. found that the change in the cervical length after 24 h did not significantly improve the sensitivity, specificity, positive and negative predictive value of birth within 1 week, when compared with cervical length at presentation alone [14]. However, Wagner et al. repeated the second cervical length measurement two to five days after the first cervical length and reported a significant correlation between delivery within 14 days and the difference between the two cervical length measurements [15]. This showed that perhaps there may be a role in repeating the cervical length after a few days rather than after 24 h.

Whilst measuring cervical length may be a reliable method of prediction of labour onset, it may not be available at all times of day due to logistic issues such as lack of a transvaginal ultrasound machine and a trained operator for measurements of cervical length. In comparison, biomarker tests such as the Actim Partus test may be more readily available, can be easily done within minutes, and does not require any machine or equipment for performance of the test.

### Limitations of study

The biggest limitation of this study would be its small sample size. Based on this study’s results, the difference in the mean first cervical length for subjects who delivered within 1 week and after 1 week is 2.8mm. To be able to detect a difference of 2.8mm, we would need 110 subjects in each group with a 0.05 significance level and 80 % power. Secondly, there is a high attrition rate of 15.8 % for the second scan and 23.2 % for the third scan. This further reduced the power of the results for the subgroup analysis and the investigation into the effect of change in cervical length. Nevertheless, the results generated from our study provided some insights and could be used as possible designs for future larger-scale studies.

It is also important to note that ultrasonography is an operator-dependent modality, with intraobserver and interobserver variation. In this study, in order to minimize observer differences, the TVUS cervical length measurements were performed by 3 designated principal sonographers who had extensive experience and were accredited by The Fetal Medicine Foundation, and had followed The Fetal Medicine Foundation protocol for measurement of cervical length [6].

### Future studies

Firstly, we hypothesize that there may be a difference when measuring the TVUS cervical length of a nulliparous cervix versus a multiparous cervix. The nulliparous cervix is tubular shaped, while the multiparous cervix is cone-shaped with an external os that hangs loose [16]. Hence, there may be value in conducting future studies focussing only on nulliparous women, with a bigger study population.

Secondly, while there had been many studies conducted on the utility of biomarkers such as fetal fibronectin and phosphorylated insulin-like growth factor binding protein in prediction of preterm birth, there is still a lack of high-quality data studying the combination of these biomarkers with cervical length measurements. There have also been recent studies on cervical elastography to quantitatively measure cervical softening, which occurs prior to delivery. Perhaps a combination of tests, together with digital examination, would ultimately improve the sensitivity and specificity of accurate detection of preterm birth.

## Conclusions

In our study, in women presenting with symptoms of PTL, measuring the transvaginal ultrasound cervical length is a reliable diagnostic test for delivery within 1 week. The change in cervical length over a period of one day was not predictive. However, the results are limited by the small sample size of the study. Further studies should be conducted with a larger sample size.

## Data Availability

The datasets used and/or analysed during the current study are available from the corresponding author on reasonable request.

## Abbreviations

PTL: Preterm labour
TVUS: Transvaginal ultrasound
ROC: Receiver Operator Characteristic
AUC: Area Under Curve

## References

[1] Chawanpaiboon S, Vogel JP, Moller AB, Lumbiganon P, Petzold M, Hogan D, Landoulsi S, Jampathong N, Kongwattanakul K, Laopaiboon M, Lewis C, Rattanakanokchai S, Teng DN, Thinkhamrop J, Watananirun K, Zhang J, Zhou W, Gülmezoglu AM (2019). Global, regional, and national estimates of levels of preterm birth in 2014: a systematic review and modelling analysis. Lancet Glob Health.

[2] Iams JD. Prediction and early detection of preterm labor. Obstet Gynecol. 2003;101(2):402 – 12. doi:10.1016/s0029-7844(02)02505-x. 10.1016/s0029-7844(02)02505-x 12576267

[3] Practice bulletin no. 171: management of preterm labor. Obstet Gynecol. 2016;128(4):e155–e164. 10.1097/AOG.0000000000001711 27661654

[4] Berghella V, Saccone G (2019). Cervical assessment by ultrasound for preventing preterm delivery. Cochrane Database Syst Rev.

[5] Thain S, Yeo GSH, Kwek K, Chern B, Tan KH (2020). Spontaneous preterm birth and cervical length in a pregnant Asian population. PLoS One.

[6] The Fetal Medicine Foundation. Certificates for assessment – cervical assessment. 2020 Retrieved from: https://fetalmedicine.org/cervical-assessment-1.

[7] Hueston WJ (1998). Preterm contractions in community settings: II. Predicting preterm birth in women with preterm contractions. Obstet Gynecol.

[8] Macones GA, Segel SY, Stamilio DM, Morgan MA. Predicting delivery within 48 hours in women treated with parenteral tocolysis. Obstet Gynecol. 1999;93(3):432-6. doi:10.1016/s0029-7844(98)00412-8. 10.1016/s0029-7844(98)00412-8 10074994

[9] Utter GO, Dooley SL, Tamura RK, Socol ML (1990). Awaiting cervical change for the diagnosis of preterm labor does not compromise the efficacy of ritodrine tocolysis. Am J Obstet Gynecol.

[10] Hernandez-Andrade E, Romero R, Ahn H, Hussein Y, Yeo L, Korzeniewski SJ, Chaiworapongsa T, Hassan SS (2012). Transabdominal evaluation of uterine cervical length during pregnancy fails to identify a substantial number of women with a short cervix. J Matern Fetal Neonatal Med.

[11] Berghella V, Baxter JK, Hendrix NW (2013). Cervical assessment by ultrasound for preventing preterm delivery. Cochrane Database Syst Rev.

[12] Sotiriadis A, Papatheodorou S, Kavvadias A, Makrydimas G (2010). Transvaginal cervical length measurement for prediction of preterm birth in women with threatened preterm labor: a meta-analysis. Ultrasound Obstet Gynecol.

[13] Palacio M, Sanin-Blair J, Sánchez M, Crispi F, Gómez O, Carreras E, Coll O, Cararach V, Gratacós E (2007). The use of a variable cut-off value of cervical length in women admitted for preterm labor before and after 32 weeks. Ultrasound Obstet Gynecol.

[14] Sotiriadis A, Kavvadias A, Papatheodorou S, Paraskevaidis E, Makrydimas G (2010). The value of serial cervical length measurements for the prediction of threathened preterm labour. Eur J Obstet Gynecol Reprod Biol.

[15] Wagner P, Sonek J, Heidemeyer M, Schmid M, Abele H, Hoopmann M, Kagan KO (2016). Repeat measurement of cervical length in women with threatened preterm labour. Geburtshilfe Frauenheillkd.

[16] Baskett TF, Calder AA. Munro Kerr’s Operative Obstetrics (12th edition). 2014 Elsevier Health Sciences.

